# A Pilot Study of COVID-19 Outcomes in People Living with HIV in Tatarstan

**DOI:** 10.3390/ijms27010288

**Published:** 2025-12-27

**Authors:** Natalya Andreeva, Svetlana Moiseeva, Alisa Garipova, Venera Shakirova, Ekaterina Martynova, Ilnur Salafutdinov, Svetlana Khaiboullina, Yuriy Davidyuk, Sinothile Khuzwayo, Ntombenhle Gama, Shahlo Turdikulova, Dilbar Dalimova, Mirakbar Yakubov, Sergey Morzunov, Albert Rizvanov, Ilsiyar Khaertynova, Emmanuel Kabwe

**Affiliations:** 1 Institute of Fundamental Medicine and Biology, Kazan (Volga Region) Federal University, 420008 Kazan, Russia; natasha_andreeva_2000@mail.ru (N.A.); ignietferro.venivedivici@gmail.com (E.M.); sal.ilnur@gmail.com (I.S.); sv.khaiboullina@gmail.com (S.K.); davi.djuk@mail.ru (Y.D.); smorzunov@gmail.com (S.M.); rizvanov@gmail.com (A.R.); 2 Department of Infectious Diseases, Kazan State Medical Academy, 420012 Kazan, Russia; sgerasimova.kgma@gmail.com (S.M.); alisa-bilalova@mail.ru (A.G.); vene-shakirova@yandex.ru (V.S.); i.khaertynova@gmail.com (I.K.); 3 Department of Medical Biology and Genetics, Kazan State Medical University, 420012 Kazan, Russia; 4 Department of Biochemistry, Genetics and Microbiology, University of Pretoria, Lynnwood Road, Hatfield, Pretoria 0002, South Africa; snothilesementha81@gmail.com (S.K.); ntombenhle.gama@up.ac.za (N.G.); 5 Center for Advanced Technologies, Tashkent 100174, Uzbekistan; shahlo.ut@gmail.com (S.T.); dilbar.dalimova@gmail.com (D.D.); mirakbardan@yahoo.com (M.Y.)

**Keywords:** HIV, SARS-CoV-2, COVID-19-only, PLWH/COVID-19, immune response

## Abstract

The coronavirus disease 2019 (COVID-19) pandemic remains a significant public health threat globally with significant socio-economic impacts. People living with human immunodeficiency virus (HIV) (PLWH) have a high risk of severe outcomes of COVID-19 due to immunosuppression. Clinical manifestation of COVID-19 in HIV patients largely remains unclear. We carried out a pilot study to investigate the clinical laboratory data and circulating cytokines in PLWH infected with severe acute respiratory syndrome coronavirus 2 (SARS-CoV-2), in Tatarstan, Russia. Serum samples were collected from three groups: PLWH with COVID-19 (PLWH/COVID-19), COVID-19-only, and uninfected controls. We found an increased risk of severe COVID-19 in PLWH/COVID-19 patients compared to COVID-19-only. Four fatal cases were in PLWH/COVID-19, while there was no fatality in COVID-19-only. Pro-inflammatory cytokines, such as IL-5, IL-6, IL-9, and IL-15, were elevated in PLWH/COVID-19 compered to COVID-19-only. These preliminary findings highlight the potential for more severe COVID-19 in PLWH/COVID-19 where pro-inflammatory cytokines could play pathogenic role.

## 1. Introduction

Coronavirus disease 2019 (COVID-19) presents a global threat to public health and has a significant socio-economic impact [1]. As of March 2025, there have been more than 777 million confirmed COVID-19 cases and more than 7 million deaths worldwide [2]. Older age and comorbidities are associated with an increased risk of severe COVID-19 [3].

HIV causes immunosuppression by depleting CD4^+^ T helper cells, making patients susceptible to bacterial, fungal, parasitic, and viral infections [4]. Severe immunosuppression and inadequate antiretroviral therapy (ART) are the risk factors for opportunistic infections [5]. Currently, over 40.8 million people are living with human immunodeficiency virus (HIV) (PLWH) worldwide; of these, 3% are in Russia and 0.04% are in Tatarstan, Russia [6,7]. The healthcare for PLWH had become critical during the COVID-19 pandemic, requiring more attention to treatment of COVID-19 [8]. The Center for Disease Control and Prevention (CDC), USA, identified PLWH as being at a high risk for severe forms of COVID-19. These patients have long hospitalization and are frequently admitted to the intensive care unit (ICU). Also, they require a life-supporting mechanical ventilation, and often die due to COVID-19-related complications. Notably, patients with comorbidities and acquired immunodeficiency syndrome (AIDS) are facing a higher risk of severe forms of COVID-19 [9].

Also, PLWH and infected with severe acute respiratory syndrome coronavirus 2 (SARS-CoV-2) have various symptoms ranging from moderate to severe forms of COVID-19 [10]. However, consensus information on the association of HIV with adverse outcomes of COVID-19 remains limited [11]. Data on the severity of COVID-19 in PLWH are inconsistent [9,12]. Some, based on a limited sample size, have suggested that HIV has a limited effect on the outcome of COVID-19 [13]. Likewise, HIV infection does not affect the course of COVID-19 [14,15,16]. However, Wang et al. reported a case of PLWH with COVID-19 (PLWH/COVID-19) where they found longer COVID-19 duration and low antibody titer [17]. In addition, PLWH/COVID-19 patients have more frequent complications compared to COVID-19-only [12,18].

In previous studies, HIV contribution to the emergence new variants of SARS-CoV-2 was suggested. For example, the emergence of the Omicron variant in southern Africa was linked to HIV-associated immunosuppression [19]. It was suggested that prolonged COVID-19 infection in an immunocompromised PLWH led to the emergence of the Omicron variant of SARS-CoV-2 [20]. Also, COVID-19 patients with advanced HIV were found to have multiple variants of SARS-CoV-2, and virus evolution continued through the course of the infection [13]. These patients had significantly reduced numbers of CD4^+^ T cells and an increased prevalence of SARS-CoV-2 variants, compared to patients without HIV who had higher numbers of CD4^+^ T cells [13]. Thus, PLWH/COVID-19 patients could have a different course of SARS-CoV-2 infection compared to SARS-CoV-2 only.

The clinical course of COVID-19 in PLWH remains poorly characterized. Our knowledge is limited particularly in understanding of the molecular mechanisms of COVID-19 in these patients. To address this gap of knowledge, we analyzed serum lipids and cytokines in PLWH/COVID-19, patients with COVID-19-only, and controls. These biomarkers reflect immune dysregulation, such as hyperinflammation and metabolic dysfunction, which influence the disease progression. Notably, HIV-associated dyslipidemia characterized by low high-density lipoprotein (HDL) and high low-density lipoprotein (LDL) in PLWH could exacerbate endothelial injury and risk of thrombosis, which can worsen COVID-19 in PLWH/COVID-19. Given the limited and inconsistent data on COVID-19 in PLWH/COVID-19 patients, we conducted a pilot study to define the clinical, biochemical, and immunological characteristics of a PLWH/COVID-19 cohort, in Tatarstan, Russia.

## 2. Results

### 2.1. Screening Samples for HIV and SARS-CoV-2 RNA

Total RNA was collected from 18 patients (9 PLWH/COVID-19 and 9 COVID-19-only). Anti-HIV antibodies were detected in all nine PLWH/COVID-19 patients by ELISA; however, HIV RNA was found only in five patients, by RT-PCR. The absence of HIV RNA in the remaining four patients could be attributed to ART treatment. SARS-CoV-2 RNA was found in all 18 patients using nested-PCR.

**Figure 1:**
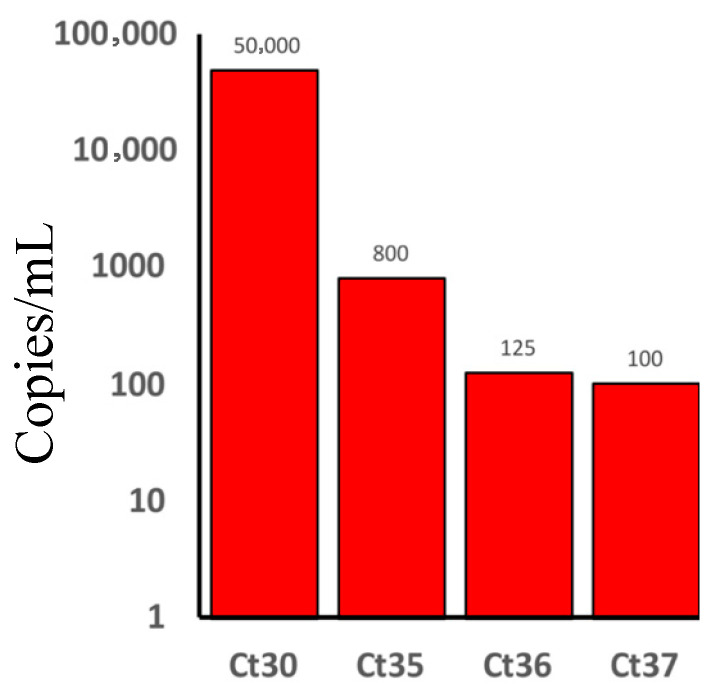
Human immunodeficiency virus (HIV) viral load in people living with HIV and had Coronavirus disease 2019 (COVID-19) (PLWH/COVID-19). The number of HIV copies/mL is presented on the y-axis (50,000 copies/mL for two patients, 800 copies/mL for one patient, 125 copies/mL for one patient, and 100 copies/mL for one patient). Threshold cycle (Ct) and the numbers represent the qPCR Ct values for each tested sample from which the viral load was calculated.

The HIV viral load was determined using qPCR in 5 PCR-positive samples. Based on World Health Organization (WHO) consolidated guidelines on HIV prevention, testing, treatment, service delivery, and monitoring: recommendations for a public health approach, two samples had a moderate HIV viral load, with 50,000 copies/mL. In the other three samples, low HIV viral load (one had 800 copies/mL, another one had 125 copies/mL, and the other one had 100 copies/mL) were found (Figure 1).

### 2.2. Phylogenetic Analysis of HIV

**Figure 2:**
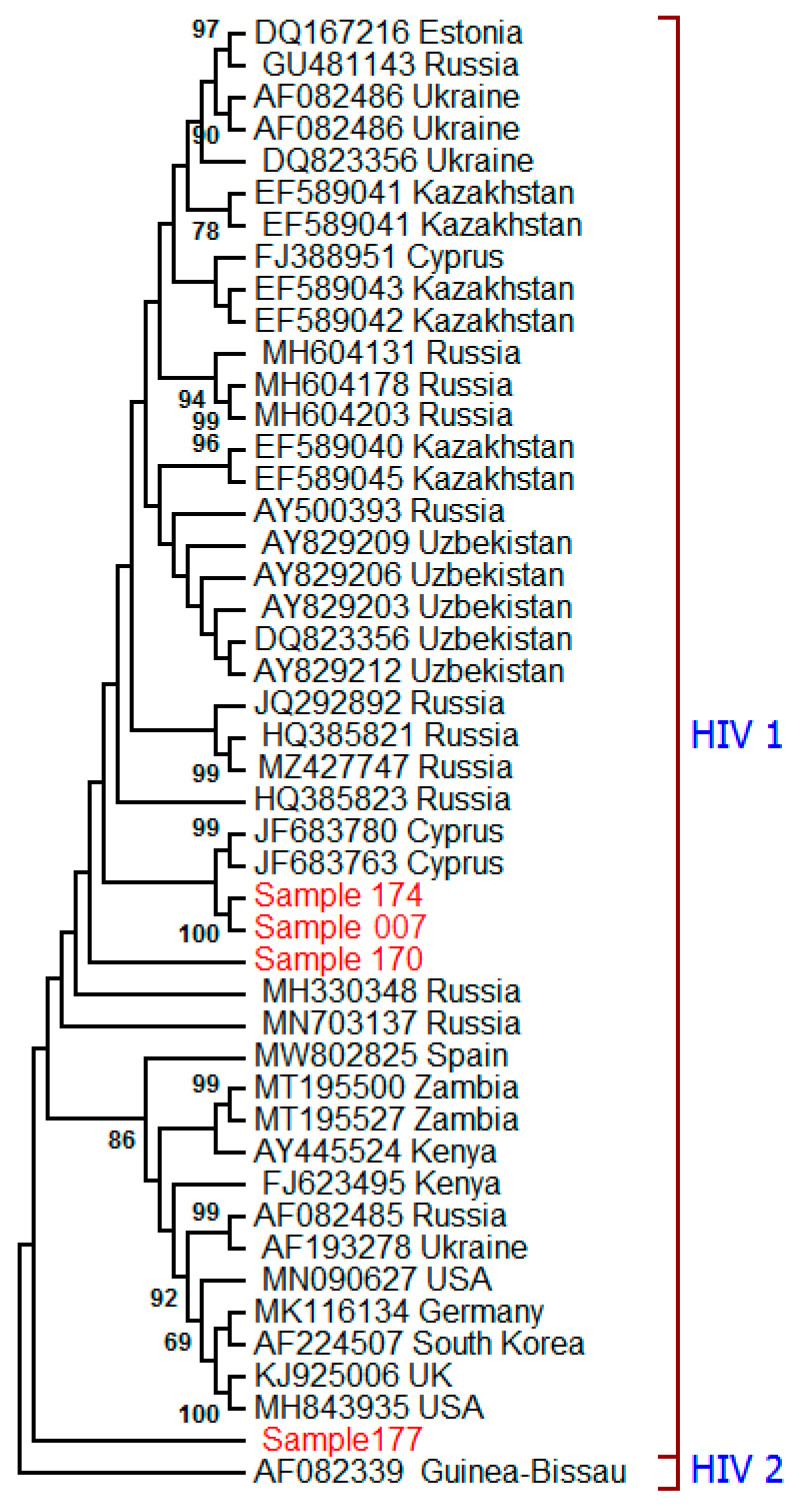
The phylogenetic tree of HIV was constructed based on the partial *Env* gene nucleotide sequence (650 bp) obtained from PLWH/COVID-19 patients. The maximum likelihood method was used based on the Tamura–Nei model in the MEGA v6.0 software [21]. The percentages of replicate trees in which the associated taxa clustered together in the bootstrap test (1000 replicates) are shown next to the corresponding branch nodes. Only values > 60% are displayed [3]. The strains marked in red are identified in this study. Other HIV-1 strains were downloaded from the GenBank database. The HIV-2 *Env* sequence from Guinea-Bissau was used as an outgroup.

The *Env* gene nucleotide sequences were obtained from four HIV-positive samples. The HIV genotypes were compared to the existing genotypes to better understand their evolutionary relationships and transmission patterns. The phylogenetic tree was constructed using 650 nucleotide amplified fragments of the *Env* (coding sequences) CDS sequences (Figure 2). Nucleotide sequences of the HIV-2 *Env* gene from Guinea-Bissau were used as an outgroup. Samples 174 and 007 clustered with Cyprus HIV-1 genotypes on the branch that contained the Russian genotypes. Also, sample 170 is very close to sample 174 and 007 on the tree. Interestingly, sample 177 did not cluster with the analyzed samples, as though it shared a clade with genotypes from Russia and other neighboring countries (Figure 2).

### 2.3. Clinical Presentation of SARS-CoV-2 Infection in PLWH/COVID-19 and COVID-19-Only

Five PLWH/COVID-19 and seven COVID-19-only patients were diagnosed with a moderate form of the SARS-CoV-2 infection when hospitalized. The remaining six patients, four PLWH/COVID-19 and two COVID-19-only, developed severe COVID-19 infection. All patients with moderate infection (5 PLWH/COVID-19 and 7 COVID-19-only) had a fever < 38 °C. The extent of lung injury, examined by computer tomography (CT), was stage 1–2. The oxygen saturation level was >93%.

Severe cases (4 PLWH/COVID-19 and 2 COVID-19-only) had fever (38.92 ± 0.66 °C) with a duration of 6.31 ± 4.04 days. The extent of lung injury was stage 3–4, and the oxygen saturation level was <90%. Also, several patients had hypotension (90/60 mmHg). Lung injury was ranked as <0% in four PLWH/COVID-19 and 20–40% in two COVID-19-only. Four severe PLWH/COVID-19 and one severe COVID-19-only required artificial ventilation and were admitted to the intensive care unit (ICU). All four ICU PLWH/COVID-19 (3 female and 1 male) died, while COVID-19-only patient in ICU was discharged.

### 2.4. Clinical Laboratory Test Results

**Figure 3:**
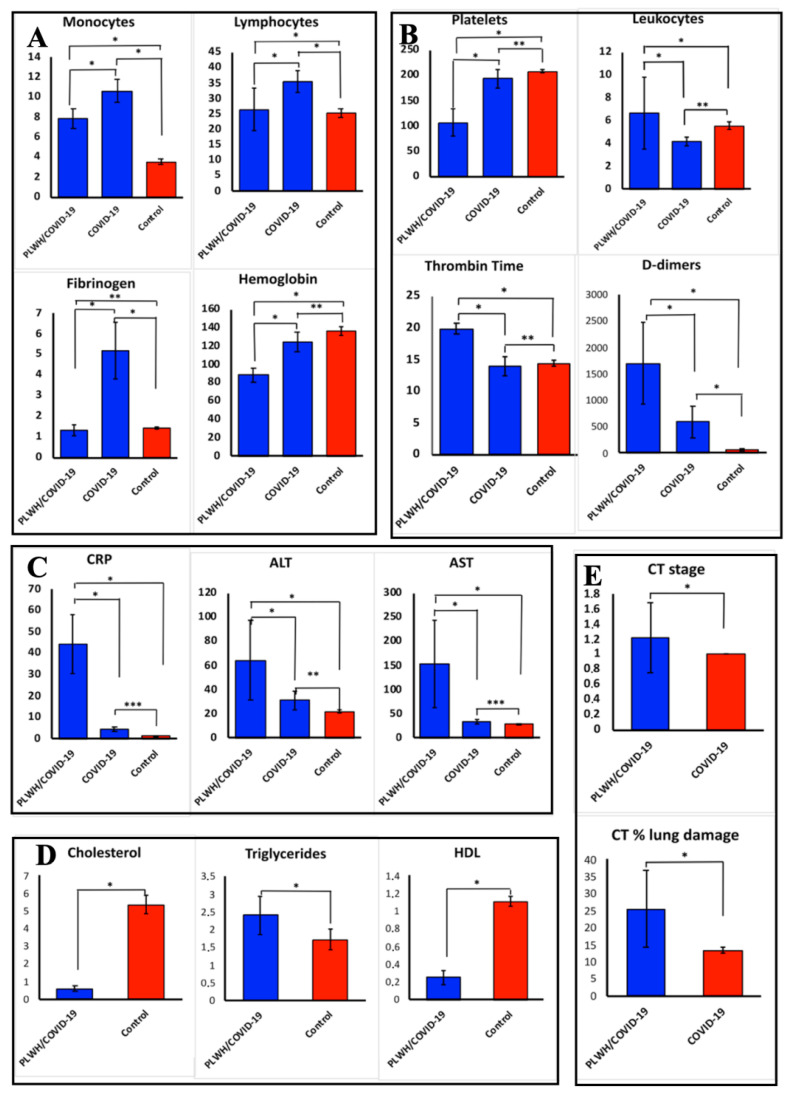
Clinical laboratory results for PLWH/COVID-19 and COVID-19-only compared to controls. (**A**–**C**)—clinical laboratory data of PLWH/COVID-19 and COVID-19-only compared to control groups. (**E**)—computed tomography (CT) stage in patients with PLWH/COVID-19 and COVID-19-only. (**D**)—clinical laboratory data in PLWH/COVID-19 and control. Each sample was analyzed in triplicate. Data are presented as median ± interquartile range (IQR). The data were not normally distributed as assessed by the Shapiro–Wilk normality test. Therefore, we used non-parametric statistical tests such as Kruskal–Wallis for all subsequent analyses, which is more appropriate for such data as they do not assume normality. Asterisks (*) indicate statistically significant differences between parameters tested ((*) *p* < 0.05, (**) *p* < 0.01, (***) *p* < 0.001, Kruskal–Wallis test).

COVID-19-only had high monocytes, lymphocyte counts, and elevated fibrinogen (Figure 3A). The PLWH/COVID-19 had decreased platelet count and leukocytosis (Figure 3B). Also, coagulopathy was evident in PLWH/COVID-19 with high levels of fibrin D-dimers and increased activated partial thrombin time (Figure 3B). Increased C-reactive protein (CRP), alanine aminotransferase (ALT), and aspartate aminotransferase (AST), indicating liver injury, were present on admission in all PLWH/COVID-19 (Figure 3C). Also, PLWH/COVID-19 had elevated triglycerides and decreased cholesterol and HDL compared to patients with COVID-19-only (Figure 3D). A computed tomography (CT) scan demonstrated subarachnoid bleeding in PLWH/COVID-19 (Figure 3E).

### 2.5. Analysis of Serum Cytokines in PLWH/COVID-19 and COVID-19-Only

**Figure 4:**
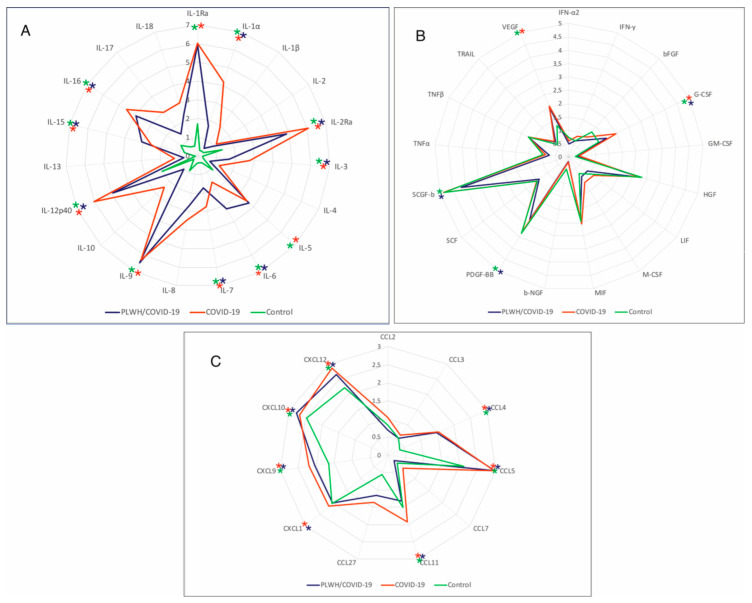
Analysis of serum cytokines in PLWH/COVID-19 and COVID-19-only patients compared to controls. (**A**) Interleukins in PLWH/COVID-19, COVID-19, and control. (**B**) Cytokines in PLWH/COVID91, COVID-19-only, and control. (**C**) Chemokines in PLWH/COVID-19, COVID-19-only, and control. Serum samples were quantified using Bio-Plex Pro Human Cytokine 48-plex Screening Panel (12007283, BioRad, Hercules, CA, USA). Each sample was tested in triplicate, and results are expressed as log_2_ fold change (Log_2_FC) relative to the control group. Data were analyzed using MasterPle CT control software and MasterPlex QT analysis software (MiraiBio, San Bruno, CA, USA). Further, statistical analysis was performed using the Kruskal–Wallis test with Benjamini–Hochberg correction for multiple comparisons. *—Significant difference between PLWH/COVID-19 and COVID-19-only; *—significant difference between COVID-19-only and control; *—significant difference between PLWH/COVID-19 and control. Asterisks (*) indicate statistically significant differences between PLWH/COVID-19, COVID-19-only, and health controls ((*) *p* < 0.05).

Serum cytokines were analyzed in PLWH/COVID-19 and COVID-19-only with severe and moderate forms of the disease and control. Six chemokines (CXCL9, CXCL10, CCL4, CCL5, CCL27, and CXCL12) and eight interleukins (IL-1Ra, IL-2Ra, IL-3, IL-5, IL-9, IL-12p40, IL-15, and IL-16) were increased in both PLWH/COVID-19 and COVID-19-only (Figure 4A,C). Increased levels of interleukins (IL-1 α, IL-7, IL-17, and IL-18) and chemokines (CCL11, and CXCL1) were found only in the COVID-19-only, whilst PLWH/COVID-19, had increased levels of four different interleukins (IL-5, IL-6, IL-9, and IL-15) and one chemokine (CXCL10) compared to COVID-19-only. Interestingly, CXCL10 was not increased in COVID-19-only. Also, G-CSF and VEGF were increased in COVID-19-only and PLWH/COVID-19 compared to the control.

## 3. Discussion

In this pilot study, including nine PLWH/COVID-19 and nine COVID-19-only, we found more severe clinical presentation and distinct circulating pro-inflammatory cytokines in SARS-CoV-2-infected PLWH. To ensure that these immunological and clinical differences were triggered by HIV strains commonly circulating in Russia, we completed a phylogenetic analysis of the HIV *Env* gene nucleotide sequences obtained from PLWH/COVID-19. It is demonstrated that HIV-1 circulates in Tatarstan, Russia. Four HIV sequences identified in this study clustered with the most prevalent HIV-1 A (315/332) subtype detected in Russia [22]. HIV-1 A is the second most dominant subtype after HIV-1 C, circulating globally [23]. Although there is limited information on the unique clinical characteristics of each HIV-1 subtype, it is known that clinical presentation and disease outcome overlap and are modified by host genetics, pathogens, and environmental factors [24]. HIV-1 A suppresses the immune system, thereby affecting the outcome of COVID-19 infection [25]. Therefore, the observed clinical differences in our studied cohort are likely attributed to infection with HIV-1 A subtype.

Our study demonstrated limited connection between HIV viral load and fatality in PLWH/COVID-19. COVID-19 severity varied, with poor outcomes observed more frequently in patients with moderate HIV viral load (50,000 copies/mL) compared to those who had lower HIV viral load (100–125 copies/mL) (Figure 1). It is worth noting that the pathological implications of low HIV viral load (100–125 copies/mL) differ significantly from those with high (50,000 copies/mL) HIV copies. Yang et al. showed that PLWH with unsuppressed HIV were more likely to have severe COVID-19 [26]. Also, Miller et al. found that low HIV load in PLWH was not linked to severe COVID-19 [27]. Therefore, whether HIV viral load contributes to COVID-19 outcomes remains unknown. While our data suggest a trend where higher HIV load correlates with worsened outcomes, the small sample size limits a definitive conclusion. The limited PLWH/COVID-19 sample size could be a result of the low prevalence of HIV in Tatarstan (394.7/100,000 population) in October 2025. Also, a contributing factor is that samples were collected during the pandemic, where PLWH were following the quarantine restrictions as they had immune-compromised condition. As a result, there was a low number of patients hospitalized with HIV during the COVID-19 quarantine.

Studies suggest that HIV patients have an increased risk of hospitalization for COVID-19 [9]. In this pilot study, we found that the PLWH/COVID-19 had a more severe form of SARS-CoV-2 infection compared to COVID-19-only. There were four fatal cases among nine PLWH/COVID-19 (44%), indicating a more severe course of infection. In contrast, COVID-19-only patients had 0 deaths out of 9 cases (0%). The extent of lung injury in PLWH/COVID-19 and COVID-19-only severe cases was in stages 3–4, and the oxygen saturation level was <90%. Interestingly, there were no fatal cases among COVID-19-only patients. It should be noted that studies have shown that comorbidity could contribute to a more severe form of COVID-19 infection, which was demonstrated in several studies [9,28,29]. Our data support this observation as we found more severe forms of COVID-19 patients with HIV comorbidity.

SARS-CoV-2 could cause abnormally high cytokine production, which is known as a “cytokine storm” [30]. Studies have found that an inflammatory cytokine signature could predict COVID-19 severity and survival [31,32,33]. In our study, interleukins (IL-1 α, IL-7, IL-17, and IL-18) and chemokines (CCL11 and CXCL1) were found increased in COVID-19-only. Also, in PLWH/COVID-19, serum levels of a different set of interleukins (IL-5, IL-6, IL-9, and IL-15) and chemokine CXCL10 were upregulated. Increased levels of these cytokines could suggest the activation of the immune response in PLWH/COVID-19 and COVID-19-only. Moreover, these cytokines are linked to the activation of cell-mediated immune response [34]. However, activation of different set of interleukins and chemokines suggests diverse immune response in these two groups of patients.

In PLWH/COVID-19, elevated IL-6, IL-5, IL-9, and IL-15 interleukins compared to COVID-19-only could induce a strong immune response in these patients. Studies have demonstrated that activation of pro-inflammatory cytokines and immune dysregulation are linked to the severity of disease and death in PLWH/COVID-19, even in patients received ART [35,36]. Elevated IL-6 could suggest an exaggerated inflammatory immune response caused by both SARS-CoV-2 and HIV. IL-15 could support the functions of NK and T cells; however, the long-term activation of this cytokine could lead to immune exhaustion [37]. The correlation between IL-15 and increased HIV-1 viremia, as well as markers of inflammation, was reported by Swaminathan et al. [38]. The high level of IL-6 and IL-15 in PLWH/COVID-19 compared to COVID-19-only may produce an synergistic effect of pre-existing HIV-related immune activation and the acute immune response induced by SARS-CoV-2. This assumption is supported by Alexandra et al., indicating that high levels of IL-5 in PLWH/COVID-19 contributed to immune dysregulation [23]. However, there are limited data on the role of high IL-5 and IL-9 in PLWH/COVID-19 in COVID-19 outcome. Further studies using a larger patient cohort are needed to validate the role of cytokines in the immune response in PLWH/COVID-19 patients.

Our pilot study also revealed significant metabolic and biochemical changes in PLWH/COVID-19, which may explain the difference in clinical presentation of SARS-CoV-2 infection. PLWH/COVID-19 had dyslipidemia, characterized by elevated triglycerides and reduced cholesterol and HDL levels compared to controls (Figure 3D). Feeney and Mallon reported similar lipid abnormalities leading to HIV metabolic syndrome [39]. Low HDL has been linked to impaired immune function and increased cardiovascular risk in PLWH [40], which could increase the severity of COVID-19. Additionally, multi-organ involvement as revealed by coagulopathy (elevated D-dimers) and liver injury (increased ALT/AST) was observed in PLWH/COVID-19. Leukocytosis and thrombocytopenia could suggest systemic inflammation and endothelial dysfunction, which are both signs of severe COVID-19. The increased CRP together with elevated IL-6 and IL-15 in PLWH/COVID-19 could indicate hyperinflammation, further explaining the observed differences in the outcome of the COVID-19. Thus, biochemical changes suggest SARS-CoV-2 infection could cause severe form of COVID-19 in PLWH due to high pro-inflammatory cytokine level, dyslipidemia, and disturbed hemostasis as compared to COVID-19-only.

Overall, our findings are in agreement with prior studies demonstrating that SARS-CoV-2 and SARS-CoV-2 trigger systemic inflammation and immune activation [35,36]. The elevation of IL-5, IL-6, and IL-15 in PLWH/COVID-19 mirrors previously reported cytokine signatures associated with hyperinflammation and adverse clinical outcomes in both infections [31]. These results extend existing observations by highlighting that, even in patients receiving ART, persistent immune activation may predispose PLWH to severe COVID-19 outcomes.

## 4. Materials and Methods

### 4.1. Study Participants and Sample Collections

Study participants: Patients were enrolled from Agafonov Republican Clinical Hospital for Infectious Disease, Tatarstan, Russia, during the pandemic, 2021. All patients had confirmed COVID-19 by detection of SARS-CoV-2 virus in nasal swabs. COVID-19-only patients were enrolled from the same hospital during 2021. Both PLWH/COVID-19 and COVID-19 patients were hospitalized at the same time, and samples were collected during their stay in the healthcare facility. Control samples were collected from individuals who did not have symptoms and had no contact with COVID-19 patients. These controls had negative anti-HIV and anti-SARS-CoV-2 antibodies by ELISA and viral RNA by RT-PCR.

Sample collections: Disease severity was classified according to the WHO COVID-19 guidelines (https://www.who.int/publications/i/item/B09467, accessed 14 September 2025). Moderate disease had clinical signs of pneumonia (fever, cough, dyspnea) with SpO2 > 90%. Severe disease was defined as having signs of pneumonia and one of respiratory rate > 30 breaths/min, severe respiratory distress, or SpO2 < 90%. Table 1 shows the baseline characteristics of study participants. Venous blood samples were collected from nine PLWH/COVID-19 (mean age 38.0 ± 11.9 years old; 5 female and 4 male), nine COVID-19-only (mean age 47.0 ± 12.6 years old; 6 female and 3 male), and nine controls (mean age 47.1 ± 13.7 years old; 3 female and 6 male) enrolled participants. The serum was separated and stored at −80 °C. The diagnosis of SARS-CoV-2 infection was made based on the clinical presentation, ELISA, and confirmed by RT-PCR.

### 4.2. Ethics Statement

The Ethics Committee of the Kazan Federal University approved this study, and signed informed consent was obtained from each patient and healthy subjects, by the methodological recommendations concerted up within the framework of the protocol (protocol 27 of the meeting of the ethics committee of the KFU dated 28 December 2020). The sampling in 2021 was carried out according to the protocol approved by the Expert Commission of the Kazan Federal University (Article 20, Federal Law “On the Protection of the Health of Citizens of the Russian Federation” No. 323-FZ dated 21 November 2011). Informed consent was obtained from each subject according to the guidelines approved under this protocol (Article 20, Federal Law “Protection of Health Rights of Citizens of Russian Federation” N323-FZ, 21 November 2011).

### 4.3. Extraction of Total RNA

Total RNA from venous blood was extracted using TRIzol reagent (Invitrogen, Carlsbad, CA, USA). Venous blood (100 μL) was mixed with 300 μL of the TRIzol reagent, incubated at room temperature for 10 min, and centrifuged for 5 min at 15,000 rpm. Then, chloroform (60 μL) was added and incubated for 10 min, and the supernatant was separated by centrifugation for 15 min at 15,000 rpm. Isopropanol (250 μL) was added to the supernatant and incubated at −20 °C for 40 min. The pellet was separated by centrifugation for 15 min at 15,000 rpm and washed twice with 600 μL of 80% ethanol. The pellet was dried, dissolved in ddH_2_O, and stored at −20 °C for analysis.

### 4.4. RT-PCR, qPCR, and Sanger Sequencing

cDNA synthesis was completed using RevertAid Reverse Transcriptase (Thermo Fisher Scientific, Waltham, MA, USA), following the manufacturer’s recommendation. The PCR amplification of HIV *Env* was conducted using primers: forward 5′-TAGAAAGAGCAGAAGACAGTGGCAATGA-3′ and reverse 5′-CAGCAGTTGAGTTGATACTACTGGCC-3′. To detect the SARS-CoV-2 S RNA, primers forward 5′-ACTGATGCTGTCCGTGATCCACAG-3′ and reverse 5′-AGCCCCTATTAAACAGCCTGCACG-3′ were used. PCR reactions were conducted using reaction mix 5×Screen Mix (Evrogen, Moscow, Russia) as instructed by the manufacturer.

The HIV viral load was determined using qPCR. Primers for qPCR were forward the same as described above and reverse 5′-TTTAGCATCTGATGCACAAAATAG-3′). Standard curves were generated using known DNA concentrations. To determine the HIV subtype, the HIV PCR amplicons were sequenced using ABI PRISM 310 and Big Dye Terminator 3.1 Sequencing kit (ABI, Waltham, MA, USA) following the manufacturer protocol. The HIV partial sequences of 650 nucleotides in length were analyzed.

### 4.5. Enzyme-Linked Immunosorbent Assay (ELISA)

Lipid concentration in the serum was determined: cholesterol, triglycerides, and HDL-C were determined using a Cholesterol commercial kit (Novosibirsk-Novo) (Vektor-Best, Novosibirsk, Russia), Triglycerides commercial kit (Novosibirsk-Novo) (Vektor-Best, Novosibirsk, Russia), and LDL Cholesterol commercial kit (Novosibirsk-Novo) (Vektor-Best, Novosibirsk, Russia), respectively. Data were obtained using a semi-automatic analyzer Infinite M200 PRO (Tecan, Mount Waverley, VIC, Australia), at 37 °C and 650 nm as per the manufacturer’s instructions. Calibrations were performed using α-D-glycerol (2.29 mmol/L) calculated as triolein, certified against standard from commercial kit Vektor-Best (Novosibirsk, Russia).

### 4.6. Multiplex Cytokine Analysis

Multiplex kit Bio-Plex Pro Human Cytokine 48-plex Screening Panel (12007283, BioRad, Hercules, CA, USA) was used to analyze serum cytokines. Per 50 uL serum aliquots, a minimum of 50 beads were counted. MAGPIX analyzer (Luminex, Austin, TX, USA) was used to collect median fluorescence intensity. Each sample was analyzed in triplicate to avoid sample bias. Preliminary data analysis was conducted with MasterPlex CT control software version 3 (MiraiBio, San Bruno, CA, USA, https://www.miraibio.com/, accessed on 15 January 2025) and MasterPlex QT analysis software version 3 (MiraiBio, San Bruno, CA, USA, https://www.miraibio.com/, accessed on 15 January 2025), and standard curves were produced using standards provided by the manufacturer. Thirty seven (37) cytokines (IL-1β, IL-2, IL-1 α, IL-1Ra, IL-2Ra, IL-3, IL-4, IL-5, IL-6, IL-7, IL-8, IL-9, IL-10, IL-12p40, IL-13, IL-15, IL-16, IL-17, and IL-18LIF, GM-CSF, IFN-α2, IFN-γ, bFGF, G-CSF, GM-CSF, HGF, LIF, M-CSF, MIF, b-NGF, PDGF-BB, SCF, SCGF-b, TNF- β, TRAIL, and VEGF) were analyzed. Additionally, serum levels of 12 chemokines (M-CSF, CCL2, CXCL9, CCL3, CCL4, CCL5, CCL7, CCL11, CCL27, CXCL1, CXCL10, and CXCL12) were measured.

### 4.7. Statistical Analysis

Statistical analyses were completed in the R environment (R-project, version 4.0.3, 2020, https://www.r-project.org/, accessed 1 November 2020). The distribution of all quantitative variables was analyzed using the Shapiro–Wilk normality test. As most parameters were not normally distributed, non-parametric methods were used for analyses. Differences between groups were assessed using the Kruskal–Wallis test, and *p*-values were adjusted for multiple comparisons using the Benjamini–Hochberg (BH) false discovery rate (FDR) procedure. Statistically significant differences between comparison groups were accepted as *p* < 0.05. Asterisks in figures indicate levels of significance (*p* < 0.05, *p* < 0.01, *p* < 0.001).

### 4.8. Phylogenetic Analysis

A phylogenetic analysis of the HIV strains was completed using the maximum likelihood method based on the Tamura–Nei model in the MEGA v6.0 software [21]. The analysis included HIV-1 sequences from Russian and other countries available in the GenBank database. The HIV-2 *Env* gene nucleotide sequence from Guinea-Bissau was used as an outgroup.

In conclusion, our pilot data show that the severity of COVID-19 varies in PLWH/COVID-19 compared to COVID-19-only. PLWH/COVID-19 had a higher risk of death and advanced lung injury compared to COVID-19-only. This could be explained by the high level of pro-inflammatory cytokines, such as IL-5 and IL-6, in PLWH/COVID-19 compared to COVID-19-only. We also found dyslipidemia and signs of disturbed hemostasis more frequently in PLWH/COVID-19 compared to COVID-19-only. Our data demonstrate the molecular mechanisms of a more severe form of COVID-19 in PLWH, which is based on the presence of signs of “cytokine storm”, dyslipidemia, and disturbed hemostasis in PLWH/COVID-19.

## Figures and Tables

**Table 1:** Baseline characteristics of study participants.

Characteristic	PLWH/COVID-19 (*n* = 9)	COVID-19-Only (*n* = 9)	Controls (*n* = 9)	*p*-Value
Demographics				
Age, years (mean ± SD)	[38.0 ± 11.9]	[47.0 ± 12.6]	[47.1 ± 13.7]	>0.05
Sex, Male (*n*, %)	4 (44.4%)	3 (33.3%)	6 (66.7%)	>0.05
Comorbidities				
Hypertension (*n*, %)	4 (44.4%)	3 (33.3%)	1 (11.1%)	>0.05
Diabetes (*n*, %)	3 (33.3%)	2 (22.2%)	0 (0%)	>0.05
Obesity (BMI ≥ 30 kg/m^2^) (*n*, %)	5 (55.6%)	4 (44.4%)	0 (0%)	>0.05
HIV-specific Data				
CD4^+^ count, cells/μL (median [IQR])	[255 [110–410]]	N/A	N/A	—
HIV Viral Load				
Suppressed (<200 copies/mL) (*n*, %)	2 (22.2%)	N/A	N/A	—
Unsuppressed (≥200 copies/mL) (*n*, %)	3 (33.3%)	N/A	N/A	—
On ART (*n*, %)	9 (100%)	N/A	N/A	—
COVID-19 Severity at Admission				
Moderate (WHO Criteria) (*n*, %)	5 (55.6%)	7 (77.8%)	N/A	>0.05
Severe (WHO Criteria) (*n*, %)	4 (44.4%)	2 (22.2%)	N/A	>0.05
Co-infections				
Hepatitis B (*n*, %)	—	N/A	N/A	—
Hepatitis C (*n*, %)	—	N/A	N/A	—

Abbreviations: BMI, body mass index; IQR, interquartile range; N/A, not applicable; PLWH, people living with HIV; SD, standard deviation. Note: *p*-values are for comparison between the PLWH/COVID-19 and COVID-19-only groups (using Fisher’s exact test for categorical variables and Mann–Whitney U test for continuous variables due to small sample size). A *p*-value < 0.05 is typically considered significant.

## Data Availability

The original contributions presented in this study are included in the article. Further inquiries can be directed to the corresponding author.
